# Ovine CD14- an Immune Response Gene Has a Role Against Gastrointestinal Nematode *Haemonchus contortus*—A Novel Report

**DOI:** 10.3389/fimmu.2021.664877

**Published:** 2021-07-16

**Authors:** Kavita Rawat, Aruna Pal, Samiddha Banerjee, Abantika Pal, Subhas Chandra Mandal, Subhasis Batabyal

**Affiliations:** ^1^ Department of BioChemistry, West Bengal University of Animal and Fishery Sciences, Kolkata, India; ^2^ Department of LFC, West Bengal University of Animal and Fishery Sciences, Kolkata, India; ^3^ Department of Animal Science, Visva Bharati University, Bolpur, India; ^4^ Department of Computer Science, Indian Institute of Technology, Kharagpur, India; ^5^ Department of Parasitology, West Bengal University of Animal and Fishery Sciences, Kolkata, India

**Keywords:** CD14 molecule, in silico analysis, *Haemonchus contortus*, sheep, differential mRNA expression analysis, molecular docking, molecular phylogeny

## Abstract

CD14 (also known as the monocyte differentiation antigen) is an important immune response gene known to be primarily responsible for innate immunity against bacterial pathogens, and as a pattern recognition receptor (PRR), binds with LPS (endotoxin), lipoproteins, and lipotechoic acid of bacteria. So far very limited work has been conducted in parasitic immunology. In the current study, we reported the role of CD14 in parasitic immunology in livestock species (sheep) for the first time. Ovine CD14 is characterized as a horse-shoe shaped bent solenoid with a hydrophobic amino-terminal pocket for CD14 along with domains. High mutation frequency was observed, out of total 41 mutations identified, 23 mutations were observed to be thermodynamically unstable and 11 mutations were deleterious in nature, causing major functional alteration of important domains of CD14, an indication of variations in individual susceptibility for sheep against *Haemonchus contortus* infestations. *In silico* studies with molecular docking reveal a role of immune response against *Haemonchus contortus* in sheep, which is later confirmed with experimental evidence through differential mRNA expression analysis for sheep, which revealed better expression of CD14 in *Haemonchus contortus* infected sheep compared to that of non-infected sheep. We confirmed the above findings with supportive evidence through haematological and biochemical analyses. Phylogenetic analysis was conducted to assess the evolutionary relationship with respect to humans and it was observed that sheep may well be used as model organisms due to better genetic closeness compared to that of mice.

## Introduction

The CD14 molecule is an important pattern recognition receptor for innate immunity reported primarily against bacteria through the process of sensitization of host cells to bacteria. CD14 exists in both membranous (mCD14) and soluble (sCD14) forms, ranging from 1 to 371 (1, 2). mCD14 is GPI-anchored, whereas sCD14 form due to bypassing of GPI addition, cleavage of the GPI anchor by phospholipase D, or direct proteolytic cleavage from the cell surface (3–5). Bacterial lipopolysaccharide or LPS (endotoxin), lipoproteins, lipoteichoic acid, and other acylated microbial products are the site of action for CD14. CD14 acts through various TLR (Toll-like Receptor) signalling complexes causing ligand binding followed by induction of intracellular proinflammatory signalling cascades (6, 7). In Gram-negative bacteria, LPS-binding protein (LBP) aids in the process of binding of LPS to CD14 (8, 9), produced by liver hepatocytes (10–12), which ultimately combines to the TLR4 complex, concentrates low levels of LPS, and leads to the increase of the sensitivity of the system (9, 10, 13, 14). MD-2 is another important co-receptor which is physically associated with TLR4 (15, 16). CD14 is a catalyst in the process of transportation of LPS to the TLR4–MD-2 complex (13, 17–20). CD14 may cause opsonisation of whole bacteria and apoptotic cells (21–25) and interact and bind to certain host phospholipids (26–28), and a wide range of acylated bacterial agonists of TLR2, namely lipoteichoic acid, peptidoglycan, mycobacterial lipoarabinomannan, atypical LPSs, and lipoproteins increasing cellular inflammatory responses (29–39). Soluble CD14 induces B cell growth and differentiation as present in bovine colostrums and milk (40). CD14 is an immune response molecule against both gram negative and gram positive bacteria (such as Mycobacterium sp., Pseudomonas sp., and Staphylococcus aureus). CD14 is an important drug target for the treatment of various diseases, like sepsis (37, 38), mastitis (41), treponemiasis (42), and glomerulonephritis (43), since it has a role in TLR agonist delivery. CD14 is equally important for parasitic diseases (44–49). CD14 as an immune response gene may be utilized in various modes for disease management. These include therapeutic use of recombinant protein, gene therapy with cloned insert, evolvement of transgenic, or gene edited disease resistant animals. Genes for innate resistance may be of practical importance for studying phylogenetic relationships. Some preliminary comparisons between humans and other mammals have already been made (50, 51). Since the CD14 gene shows a considerable degree of variability among the species, it can be used as a marker for phylogenetic analysis.

Cloning and sequencing of the CD14 gene in domestic ruminants like cattle (*Bos taurus* (52), Buffalo (*Bubalus bubalis* (53), and goat *(Capra hircus* (54), have already been conducted earlier in our lab. The present investigation aims towards the cloning and sequencing of the CD14 gene in sheep (*Ovis aries*), another important domestic ruminant. Currently, only the crystal structure of mouse CD14 (unliganded) purified from SF9 insect cells (39) and human CD14 (55) are available in databases. Both mouse and human CD14 have an N-terminal hydrophobic cavity that provides a putative binding site for LPS and other acylated ligands. So, the present work objective is to computationally model the structures of CD14 in sheep using the existing experimentally determined structures of CD14 of livestock we studied earlier. Additionally, we focus on identifying and comparing the structural and functional domain of CD14 derived peptide using bioinformatics tools, and exploring the domains in 3D structure responsible for pathogen binding. The molecular phylogenetic analysis in domestic ruminants with other domestic and wild mammals and avians with respect to CD14 gene (responsible for innate resistance) and derived peptide, has also been studied which might be helpful to look into evolutionary ecology and pharmacogenomic studies. *In silico* analysis with molecular docking were conducted to detect the role of CD14 in parasitic immunity. Differential mRNA expression profiling for non-infected and infected (*Haemonchus infested*) sheep with respect to the CD14 gene was conducted to confirm the above findings.

## Materials and Methods

### Sample Collection and RNA Isolation

Sheep liver tissue (1g) was collected from a slaughter house located under Kolkata Municipality Corporation. Adult males (n=6) of about 1-1.5 years of age were selected for collection of samples. Liver tissue was immersed in Trizol in vial and transported in ice to the laboratory for RNA isolation. Total RNA was isolated using TRIzol extraction method (Life Technologies, USA), as per the standard procedure and was further utilized for cDNA synthesis (56). Since the samples were collected from the slaughter house, ethical approval was not necessary. cDNA concentration was estimated and samples above 1200 microgram per ml were considered for further study. cDNA was employed for the characterization of ovine CD14 gene.

### cDNA Synthesis and PCR Amplification of the CD14 Gene

20μL of reaction mixture was composed of 5μg of total RNA, 0.5μg of oligo dT primer (16–18mer), 40U of Ribonuclease inhibitor, 1000M of dNTP mix, 10mM of DTT, and 5U of MuMLV reverse transcriptase in appropriate buffer. The reaction mixture was mixed thoroughly followed by incubation at 37°C for 1 hour. The reaction was allowed for up to 10 minutes by heating the mixture at 70°C unliganded and then chilled on ice. Afterwards, the integrity of the cDNA was checked by performing PCR. Concentration of cDNA was estimated through Nanodrop. Ovine CD14 primer pair was designed based on the CD14 mRNA sequences of cattle (GenBank Acc No.AF141313) using DNASTAR software (Hitachi Miraibio Inc., USA) to amplify full length open reading frame (ORF) of CD14 gene sequence of sheep. The primers were Forward: CD14-1-F ATGGTCTGCGTGCCCTACCTG and Reverse: CD14-70-1- R GGAGCCCGAGGCTTCGCGTAA. 25μL of reaction mixture was comprised of 80–100ng cDNA, 3.0μL 10X PCR assay buffer, 0.5μL of 10mM dNTP, 1U Taq DNA polymerase, 60ng of each primer, and 2mM MgCl_2_. PCR-reactions were performed in a thermocycler (PTC-200, MJ Research, USA) with cycling conditions as, initial denaturation at 94°C for 3min, further denaturation at 94°C for 30sec, annealing at 61°C for 35sec, and extension at 72°C for 3min were conducted for 35 cycles followed by final extension at 72°C for 10 min.

### cDNA Cloning and Sequencing

Amplified product of ovine CD14 was checked by 1% agarose gel electrophoresis. The products were purified from gel using Gel extraction kit (Qiagen GmbH, Hilden, Germany).pGEM-T easy cloning vector (Promega, Madison, WI, USA) was used for cloning. Then, 10μL of ligated product was mixed thoroughly to 200μL competent cells, and heat shock was given at 42°C for 45sec in a water bath. Subsequently, the cells were immediately transferred on chilled ice for 5 min., and SOC medium was added to it. The bacterial culture was centrifuged to obtain the pellet and plated on an LB agar plate containing Ampicillin (100mg/mL) added to the agar plate @1:1000, IPTG (200mg/mL) and X-Gal (20mg/mL) for blue-white screening. Plasmid isolation from overnight-grown culture was carried out by small-scale alkaline lysis method as described in (54). Recombinant plasmids were characterized by PCR using CD14 primers as reported earlier and restriction enzyme digestion. CD14 gene fragments released by enzyme EcoRI (MBI Fermentas, USA) were inserted in recombinant plasmid which was sequenced by dideoxy chain termination method with T7 and SP6 primers in an automated sequencer (ABI prism, Chromous Biotech, Bangalore).

### Sequence Analysis

DNASTAR Version 4.0, Inc., USA software was employed for the nucleotide sequence analysis for protein translation, sequence alignments, and contigs comparisons. Novel sequence was submitted to the NCBI Genbank and accession number KY110723.1 was obtained which is available in the public domain. CD14 sequences have already been analysed earlier in our lab for cattle (GU368102), buffalo (DQ457089), and goat (DQ457090). **Supplementary Table 1** lists the source and accession numbers for the CD14 sequences used for the current study.

### Study of Predicted Ruminant CD14 Protein Using Bioinformatics Tools

Predicted peptide sequences of CD14 gene of sheep and other ruminants (cattle, buffalo, goat), characterized in our lab earlier, were then aligned with the CD14 peptide of other rodent species and *Homo sapiens* using MAFFT (57). The analysis was conducted for a sequence-based comparative study among ruminants with rodent and human. Signal peptide is essential to prompt a cell to translocate the protein, usually to the cellular membrane and ultimately signal peptide is cleaved to give mature protein. The prediction of presence and location of signal peptide of CD14 gene was conducted using the software (SignalP 3.0 Sewer-prediction results, Technical University of Denmark). Pattern recognition receptor as CD14 is rich in Leucine, hence it is essential to calculate leucine percentage. Leucine percentage was calculated manually from predicted peptide sequence. Di-sulphide bonds are essential for protein folding and stability, ultimately. It is the 3D structure of protein which is biological active. Di-sulphide bonds were predicted using suitable software (http://bioinformatics.bc.edu/clotelab/DiANNA/) (39).

Protein sequence level analysis was employed (http://www.expasy.org./tools/blast/) for assessment of leucine rich repeats (LRR), leucine zipper, detection of Leucine-rich nuclear export signals (NES), and detection of the position of GPI anchor, N-linked glycosylation sites. Since CD14 is a pattern recognition receptor, these are rich in leucine rich repeat, which are essential for pathogen recognition and binding. Leucine zipper is essential for the dimerization of the CD14 molecule. N-linked glycosylation is important for the molecule to determine its membranous or soluble form. Leucine rich nuclear export signal is essential for export of CD14 from nucleus to cytoplasm, whereas GPI anchor is responsible for anchoring in the case of membranous protein. Prosite was used for LRR site detection.

Leucine-rich nuclear export signals (NES) was analysed with NetNES 1.1 Server, Technical university of Denmark. O-linked glycosylation sites were detected using NetOGlyc 3.1 server (http://www.expassy.org/), whereas N-linked glycosylation site were assessed through NetNGlyc 1.0 software (http://www.expassy.org/). Sites for leucine-zipper were detected through Expassy software, Technical university of Denmark (58). Sites for alpha helix and beta sheet were detected using NetSurfP-Protein Surface Accessibility and Secondary Structure Predictions, Technical University of Denmark (59). Domain linker sites were predicted (60). LPS-binding (61) and LPS-signalling sites (62) were predicted based on homology studies with other species of CD14 polypeptide. These sites are important for pathogen recognition and binding.

### Three Dimensional Structure Prediction and Model Quality Assessment

A three dimensional model of ovine CD14 polypeptide was predicted through Swissmodel repository (63). Templates possessing the greatest identity of sequences with our target template were identified with PSI-BLAST (http://blast.ncbi.nlm.nih.gov/Blast (64). PHYRE2 server based on ‘Homology modelling approach’ was used to build a three dimensional model of ovine CD14 (65). The molecular visualization tool PyMOL (http://www.pymol.org/) was employed for model generation and visualization of three dimensional structure of ovine CD14. The structure of ovine CD14 molecule was evaluated and assessed for its stereochemical quality (through SAVES, Structural Analysis and Verification Server, http://nihserver.mbi.ucla.edu/SAVES/); then refined and validated through ProSA (Protein Structure Analysis) web server (https://prosa.services.came.sbg.ac.at/prosa) (66). NetSurfP server (http://www.cbs.dtu.dk/services/NetSurfP/ (59) was used for assessing the surface area of ovine CD14 through relative surface accessibility, Z-fit score, and probability of alpha-Helix, beta-strand, and coil score.

Alignment of 3-D structure of ovine CD14 with other ruminant species as cattle, buffalo, and goat were analysed with RMSD estimation to evaluate the structural differentiation by TM-align software (67). Thermodynamic stability of the protein with mutations and the deleterious nature of the mutant amino acid were analysed through I-mutant 2.0 (68) and Provean analysis (69) respectively.

### Protein-Protein Interaction Network Depiction

In order to understand the protein interaction network of the CD14 protein, we performed a search in STRING 9.1 database (70). The functional interaction was assessed with confidence scores. Interactions with scores of < 0.3, scores ranging from 0.3 to 0.7, and scores of >0.7 were classified as low, medium, and high confidence respectively. Also, we executed KEGG analysis which depicted the functional association of CD14 with other related proteins.

### Phylogenetic Analysis

The nucleotide sequence of Ovine CD14 was aligned with the published nucleotide sequences of other ruminant species we characterized earlier and also with other species obtained from gene bank (http://www.ncbi.nlm.nih.gov/blast) as listed in **Supplementary Table 1** for CD14. The ClusterIV method of MegAlign Programme of Lasergene Software (DNASTAR) was employed for the analysis.

### Differential mRNA Expression Profiling for the CD14 Gene With Respect to Non-Infected and Infected Sheep

The sheep population under current study was divided in two groups, infected (infected with parasitic infection of *Haemonchus contortus*) and non-infected (no infection), as assessed through egg per gram count.

### Animals, Faecal Sample Collection, and Determination of FEC

We randomly collected 60 sheep samples from LFC farm, WBUAFS, Mohanpur campus. The samples were collected prior to routine deworming procedure, presumed to have pre-existing gastro intestinal parasites since they were not dewormed for the last three months. The animals were presumed to have been exposed to natural infection during grazing.

Faecal samples of the sheep were examined thoroughly by salt floatation method and the Faecal Egg Count were screened for each and every sample. Based on statistical analysis, samples were collected from two groups, designated as non-infected ($\bar{x}$+SD, where $\bar{x}$ stands for mean and SD means standard deviation) and infected ($\bar{x}$- SD, where $\bar{x}$ stands for mean and SD means standard deviation). Sheep were regularly sold and slaughtered for the purpose of mutton production unit. Altogether 12 tissue samples were screened, six each with low FEC (grouped as healthy) and six infected samples for further case study. Confirmation of the infected animals were also done through direct visualization of adult parasite in the abomassum and through microscopic examination. Tissue samples were collected for abomassum.

Serum was harvested from blood samples as per the standard procedure. The serum biochemical parameters like glucose, total protein, albumin, globulin, uric acid, creatinine, BUN, triglyceride, cholesterol, HDL, LDL concentrations and serum enzyme variables (AST, ALT, & AP) were estimated by using a semi-auto biochemistry analyser (Span diagnostic Ltd.) with standard kits as per the process described below.

#### Collection of Blood Samples

Blood sample was collected aseptically from the jugular vein of sheep in the separate sterile vials with anticoagulants in the morning hours between 8 a.m. to 10 a.m. 2-3ml blood was used for haematological parameters, 5ml blood for serum without anticoagulant were collected centrifuged (10 min at 1000 rpm) and preserved at – 20°C until analysis for other biochemical and serological tests.

#### Haematological Profiles

The haematological parameters like hae moglobin, erythrocyte sedimentation rate (ESR) and packed cell volume (PCV) were estimated in whole blood soon after the collection of blood. Haemoglobin was estimated by acid haematin method (71), E.S.R. and PCV by Wintrobe’s tube (72). The total erythrocyte count (TEC), total leukocyte count (TLC) and Differential leukocyte count (DLC) were studied by standard methods described by Jain (73).

#### Biochemical Analysis

The serum biochemical parameters estimated in the experiments were total protein, albumin, globulin, albumin: globulin, aspartate aminotransferase (AST), alanine aminotransferase (ALT), alkaline phosphatase (ALP), Total bilirubin, Indirect bilirubin, direct bilirubin, glucose, uric acid, urea, and BUN by using a semi-auto biochemistry analyser (Span diagnostic Ltd.) with standard kits (Trans Asia Bio-Medicals Ltd., Solan, HP, India). The methodology used for estimation of total protein, albumin, total & direct bilirubin, ALT, ALP, glucose, creatinine urea, and uric acid were biuret method, bromocresol green (BCG) method, 2- 4-DNPH method, modified kind and king’s method, GOD/POD method, modified Jaffe’s Kinetic method. GLDH-urease method, and trinder peroxidise method respectively.

### Real Time PCR (qRT-PCR)

An equal quantity of cDNA as quantified through Nanodrop was used in each reaction of 96 well optical plate of ABI 7500 system. Each reaction consisted of 1ng cDNA template, 10 µl of 2X SYBR Green PCR Master Mix, 20pMol each of forward and reverse primers to make up a final volume of up to 20 µl with nuclease free water. Each sample was run in triplicate. Analysis of real-time PCR (qRT-PCR) was performed by delta-delta-Ct (ΔΔCt) method, Ct denotes the threshold value. The list of primers used for QPCR study have been listed below with annealing temperature at 60 C.

CD14 primer: F ACCACCCTCAGTCTCCGTAA,
R- GTGCTTGGGCAATGTTCAG
18S rRNA primer: F-TCCAGCCTTCCTTCCTGGGCAT,
R-GGACAGCACCGTGTTGGCGTAGA.

### *In Silico* Study for the Detection of Binding Site of CD14 With *Haemonchus contortus* With Molecular Docking

Molecular docking is a bioinformatics tool used for *in silico* analysis for the prediction of binding mode of a ligand with a protein 3D structure. Patch dock is an algorithm for molecular docking based on shape complementarity principle (74). Patch Dock algorithm was used to predict ligand protein docking for surface antigen for gastrointestinal parasitism with *Haemonchus contortus* (alpha tubulin and beta tubulin antigen) with ovine CD14.

## Result

### Cloning and Sequencing of CD14 cDNA of Sheep

**Figure 1:**
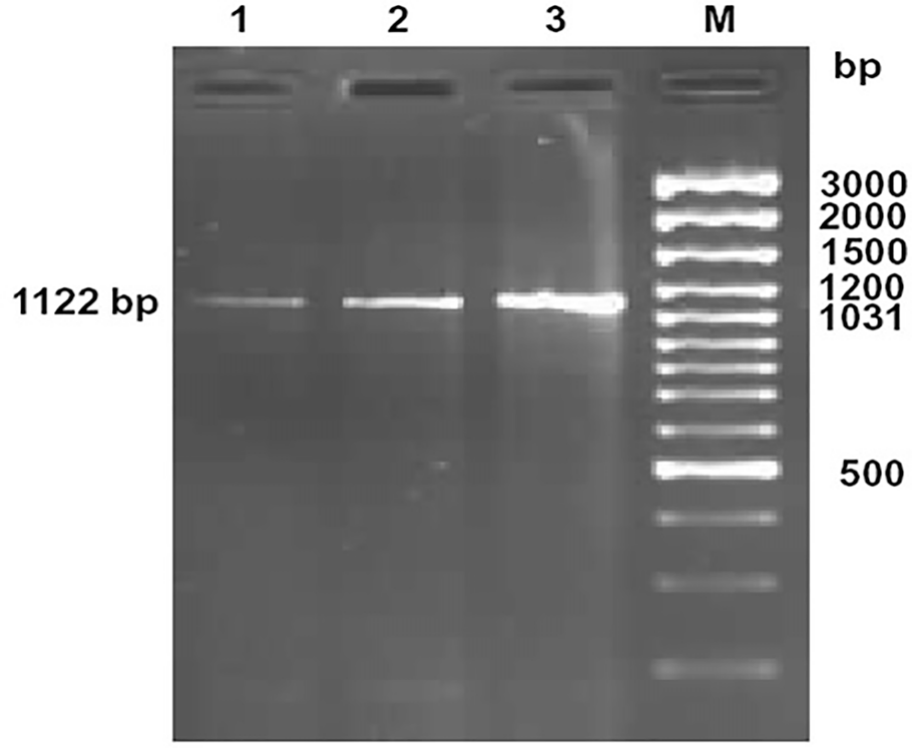
Amplification of 1122bp fragment of CD14 gene of sheep and goat. Lane 1-2: Amplified product from cDNA of CD14 gene of sheep Lane M: 100bp DNA ladder plus.

**Figure 2:**
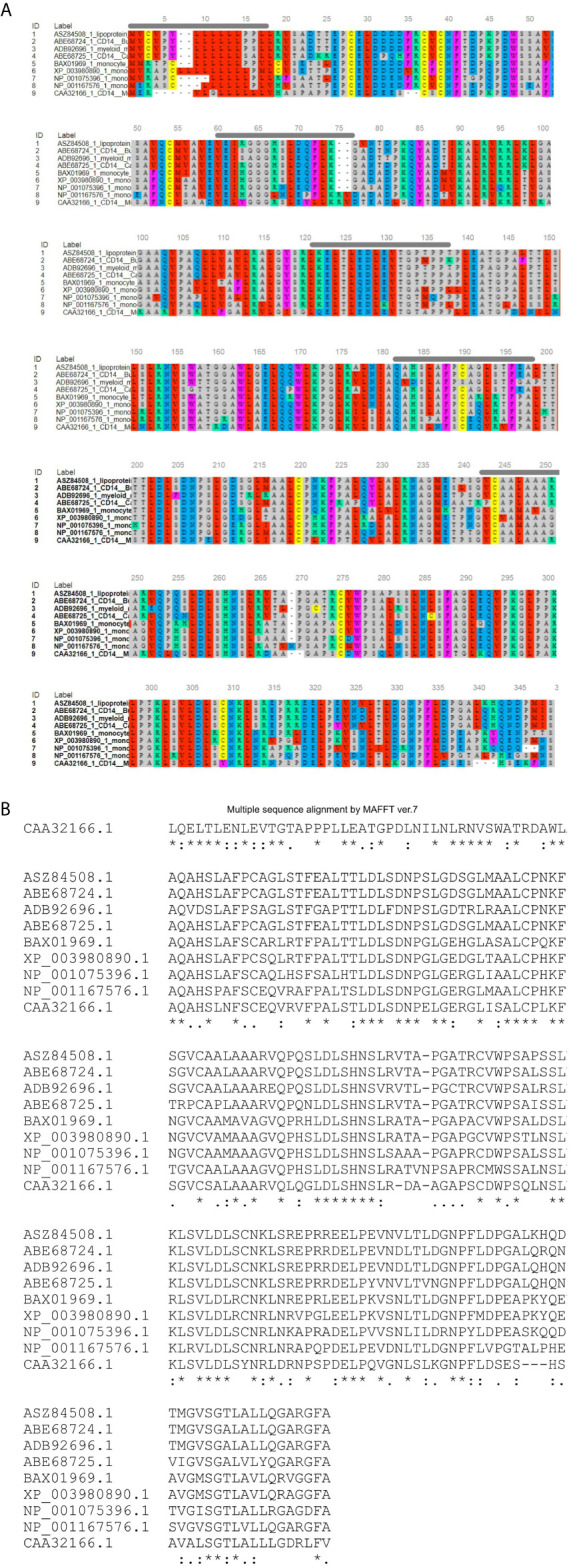
Alignment of ruminant CD14 derived peptide with other species. **(A)** Alignment through MSA viewer. **(B)** CLUSTAL format alignment by MAFFT. 1: Sheep (Ovis aries), 2: CB cattle (Bos taurus x Bos indicus), 3: Buffalo (Bubalus bubalis), 4: Goat (Capra hircus), 5: Human (Homo sapiens), 6: Mice (Mus musculus), 7: Dog (Canis lupus familiaris), 8: Cat (Felis catus), 9: Horse (Equus caballus). Green line, Signal peptide; Blue line, LPS binding site; Yellow line, LPS signaling site; Red line, Leucine zipper; Black overline, Leucine rich repeat; Red line, Leucine zipper; Pink line, Domain linker prediction site. * means consensus for single amino acid and it is conserved across the species. ** means two successive amino acids for CD14 correspond to consensus sequence and these amino acid are conserved across the species. Similarly *** corresponds to three amino acids. **** corresponds to four amino acids and so on.

The ovine CD14 was observed to have an open reading frame of 1116 nucleotide, ATG as start codon and TAA as stop codon (KY110723) (**Figure 1**). Nucleotide sequences as well as derived peptide sequences for *Bos taurus* (GU368102), *Bubalus bubalis* (DQ457089), and *Capra hircus* (DQ457090) had been sequenced earlier in our lab and submitted to the gene bank. Ovine CD14 derived peptide was observed to have 373 amino acids precursor corresponding to the coding sequence of the gene and a signal peptide consisting of 20 amino acids (**Figure 2**). The molecular weight of sheep CD14 was observed to be 39645 Dalton. Slight differences have been observed in the molecular weight of the CD14 molecule of ruminants. **Table 1** lists the amino acid variations in different species of ruminants.

**Table 1:** Amino acid variations in different species of ruminants.

Sl no.	Amino acid position	Buffalo	Cattle	Sheep	Goat	Remarks/Domain present	Analysis by I-mutant	Analysis by Provean
1	13	P	P	P	**S**	Site for signal peptide	Large decrease of stability	
2	14	P	**S**	P	**A**		Large decrease of stability	
3	22	T	T	T	**K**		Large decrease of stability	
4	23	T	T	T	**R**			
5	30	D	D	D	**P**	Domains for LPS binding site 1, LPS signaling site, site for LRR		Deleterious
6	31	D	D	D	**Q**			
7	32	D	D	D	**H**			
8	74	**A**	**A**	V	**A**		Large decrease of stability in A	
9	77	**N**	**T**	**D**	**D**	LPS binding site	Large decrease of stability N,	
10	131	**M**	T	T	T	LRR site		
11	143	**F**	L	L	L	LRR site	Large decrease of stability	
12	173	**V**	**V**	**A**	**A**			
13	179	A	**V**	A	A			
14	186	C	**S**	C	C	Site for di-sulphide bond	Large decrease of stability	Deleterious
15	193	E	**G**	E	E		Large decrease of stability	
16	195	L	**P**	L	L		Large decrease of stability	Deleterious
17	201	S	**F**	S	S			Deleterious
18	210	G	**R**	G	G			Deleterious
19	212	M	**R**	M	M		Large decrease of stability	
20	221	P	P	P	**R**		Large decrease of stability	Deleterious
21	223	L	L	L	**P**		Large decrease of stability	Deleterious
22	236	**L**	P	P	**A**		Large decrease of stability	
23	238	G	G	G	**R**			
24	239	V	V	V	**P**		Large decrease of stability	Deleterious
25	248	V	**E**	V	V		decrease of stability	
26	252	S	S	S	**N**			
27	263	A	**L**	A	A	Site for LRR	Large decrease of stability	
28	267	A	**C**	A	A		Large decrease of stability	
29	276	**L**	**L**	**P**	**I**		Large decrease of stability	
30	281	L	L	L	**C**	Domain linker, leucine zipper	Large decrease of stability	Deleterious
31	318	E	E	E	**Y**	Domain		
32	321	**D**	**D**	**V**	**V**	Linker	Large decrease of stability	
33	336	Q	Q	**K**	Q		Large decrease of stability	
34	337	**R**	H	H	H			
35	345	G	G	G	**R**	LRR		Deleterious
36	349	A	A	A	**D**	LRR	Large decrease of stability	
37	350	C	C	C	**R**			
38	353	S	**F**	S	**P**			Deleterious
39	356	T	T	T	**V**			
40	364	A	A	A	**V**			
41	366	L	L	L	**Y**		Large decrease of stability	

Letters representing different amino acids in the bold indicates the variation from the consensus sequence.

### Bioinformatics Analysis for CD14 Gene

Ovine CD14 was observed to have high GC content. The GC content of the CD14 gene in ruminants was found to be high (62.2 to 62.7%), as depicted in **Table 2**. Alignment of the derived peptide sequence of ruminants have been depicted in **Figure 4**. Amino acid cysteine contains sulphur, which is responsible for disulphide bond formation, which in turn are responsible for protein folding. The site for disulphide bond has been predicted in **Table 2**. LPS binding and LPS signalling sites are very important to determine the potentiality for innate immunity of the CD14 gene. Derived peptide of the ruminant CD14 gene differs in putative N-linked glycosylation sites at amino acid positions, four sites in cattle and buffalo and three sites in goat were predicted (**Table 2**).

**Table 2:** Bioinformatics analysis of CD14 derived peptide for domestic ruminants.

	*Bubalus bubalis*	cross of *Bos taurus X Bos indicus*	Ovis aries	*Capra hircus*,
Gene Bank Protein id	ABE68724	ADB92696.1	ASZ84508.1	ABE68725.1
GC content of gene	62.3	62.7	62.92	62.21%.
leucine rich repeats	Six	Nine	Three	Seven
leucine content,	17.2%	16.89%	16.89%	15.5%
putative N-linked glycosylation sites,	4	4	4	3
sites for O-linked glycosylation	***3 sites***	5 sites for O- linked glycosylation at amino acid positions 128, 131, 134, 145, 147,	6 sites	5 O-linked glycosylation sites. amino acid positions 128, 131, 139, 144 and 145
			Thr 13,17,19,21	
			131, 237,	
disulphide bridges	***Five***	Four	Four	Four
		amino acid positions 26-37, 39-52, 186-216, 240-270		
LPS binding site	4 sites,	four LPS binding sites were depicted from 29^th^ -32^nd^, 44^th^-47^th^, 55^th^-59^th^ and 77^th^ -83^th^ amino acid positions	four LPS binding sites were depicted from 29^th^ -32^nd^, 44^th^-47^th^, 55^th^-59^th^ and 77^th^ -83^th^ amino acid positions	Four LPS binding site from 29^th^ -32^nd^, 44^th^-47^th^, 55^th^-59^th^ and 77^th^ -83^th^ amino acid positions
	29^th^ -32^nd^, 44^th^-47^th^, 55^th^-59^th^ and 77^th^ -83^th^ amino acid positions			
LPS signaling sites	Three LPS signalling sites were predicted for amino acid positions as 27^th^ -33th,109-119^th^, 169-171th	Three LPS signalling sites were predicted for amino acid positions as 27^th^ -33th,109-119^th^, 169-171th amino acid	Three LPS signalling sites were predicted for amino acid positions as 27^th^ -33th,109-119^th^, 169-171th amino acid	three LPS signaling sites
				Three LPS signalling sites were predicted for amino acid positions as 27^th^ -33th,109-119^th^, 169-171th
regions of alpha helix	***7 regions of alpha helix from amino*** acid 4-12, 46-52, 65-75, 186-190, 241-244, 335-337 and 361-367and	five regions were detected for alpha helix conformation as 4-12, 67-73, 241-244, 343-346, 360-368		five regions of alpha helix amino acid positions 4-18, 46-52, 81-84, 241-244 and 361-370
regions of beta strand	11 regions for beta strands were predicted from amino acid positions 119-121, 145-147, 173-175, 181-182, 197-199, 225-227, 252-254, 278-280, 299-301, 321-323 and 345-347 in bubaline CD14 molecule	eleven regions were identified for beta strand conformation as 60-61, 118-122, 145-147, 173-176, 181-182, 197-199, 225-227, 252-254, 278-280, 299-301, 321-324 amino acid positions		twelve regions of beta sheet were predicted
				amino acid positions 26-27, 34-37, 59-60, 118-122, 145-147, 173-176, 181-182, 197-199, 225-228, 252-254, 299-301, 321-323
, leucine rich nuclear export signal	7 detected at amino acid positions 12, 15, 16, 17,117, 122, 127	7 sites at amino acid positions 12, 15, 16, 17, 117, 122,127	7 sites at amino acid positions 12, 15, 16, 17, 117, 122,127	seven amino acid positions. acid positions 15, 16, 17, 20, 117, 122, and 127 of goat CD14 peptide
leucine zipper	amino acid position 279^th^ to 300^th^ in bubaline CD14 gene	amino acid position 279 of CB CD14 peptide	amino acid position 279 of CB CD14 peptide	Leucine zipper pattern was found at amino acid position 279
domain linker,	Domain linker site was predicted for amino acid positions 121-146 and 283-334	for amino acid positions 121-146 and 283-334	for amino acid positions 121-146 and 283-334	Domain linker site was predicted for amino acid positions 121-150 and 283-334.
site for a glycosyl phosphatidyl inositol anchor located at C-terminus	glycosyl phosphatidyl inositol (GPI) anchor located at C-terminus near 353th position of the CD14 molecule	glycosyl phosphatidyl inositol (GPI) anchor located at C-terminus near 345^th^ position of the CD14 molecule	glycosyl phosphatidyl inositol (GPI) anchor located at C-terminus near 353th	glycosyl phosphatidyl inositol (GPI) anchor located at C-terminus near 344th position of the CD14 molecule
Molecular weight	39705.07 Daltons	39939.29 Daltons	39645.00	39929.42 Da

Writings in bold indicates the variation in a particular species with respect to other species under comparison.

O-linked glycosylation site for ruminant CD14 differs, three in Buffalo and five in both goat and crossbred cattle (**Table 2**), but absent in human (32). O-linked Glycosylation is needed when hydrophilic clusters of carbohydrates alter the polarity and solubility of protein or protein folding. CD14 and other Toll- like receptors were characterized by presence of LRR and high leucine content. Leucine rich repeats (LRR) predicted peptide sequence of ruminant CD14 cDNA were studied (**Table 2** and **Figures 2A, B**). A protein dynamics study revealed that in ruminants, the position of glycosyl phosphatidyl inositol (GPI) anchor located at C-terminus of the CD14 molecule differs (**Table 2**). Seven sites for leucine rich nuclear export signal (NES) were detected in the CD14 gene of ruminants (**Table 2** and **Figures 2A, B**). In terms of secondary structure prediction, seven regions of alpha helix and eleven regions of Beta strands with different amino acid position in ruminant CD14 molecule were predicted. (**Table 2**). A leucine zipper pattern was found at amino acid position 279^th^ in the ruminant CD14 gene (**Figures 2A, B**). The site for Domain linker for ovine CD14 site was predicted, which is almost similar to amino acid positions in other ruminants (**Table 2** and **Figures 2A, B**).

**Figure 3:**
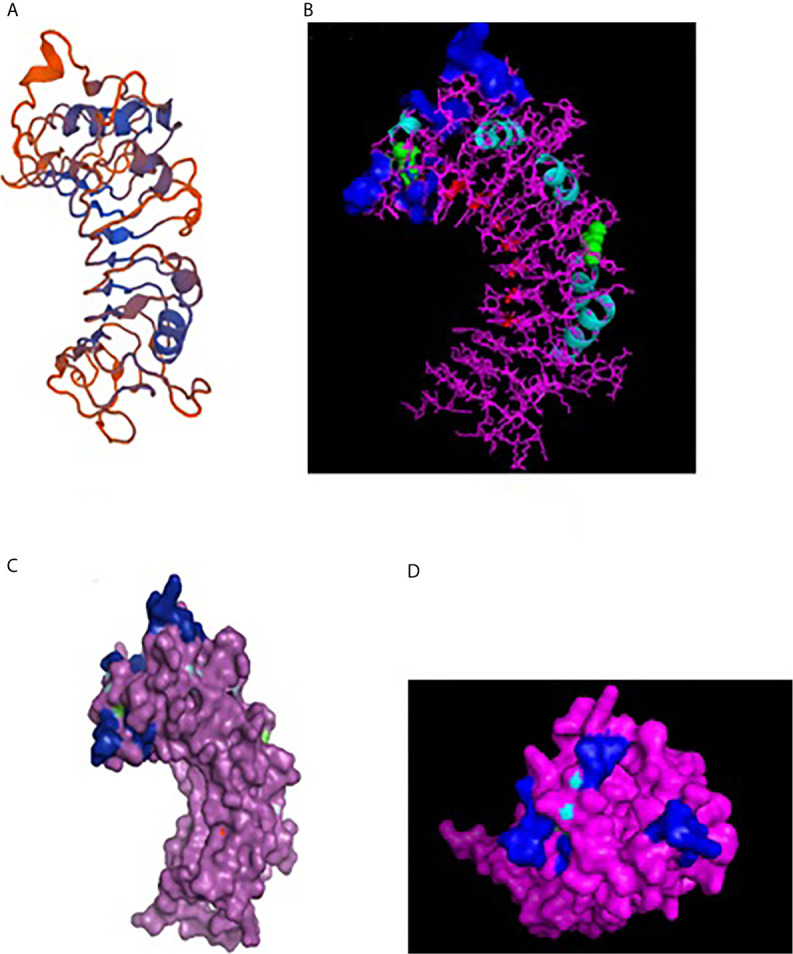
3D structure of sheep CD14. **(A)** 3D structure of ovine CD14 gene-Swiss prot view. **(B)** 3D structure of ovine CD14 gene-Pymol view (with LPS binding sites). **(C)** 3D structure of ovine CD14 gene-Surface view (with LPS binding sites). **(D)** Sheep CD14- groove (pathogen binding site).

**Figure 4:**
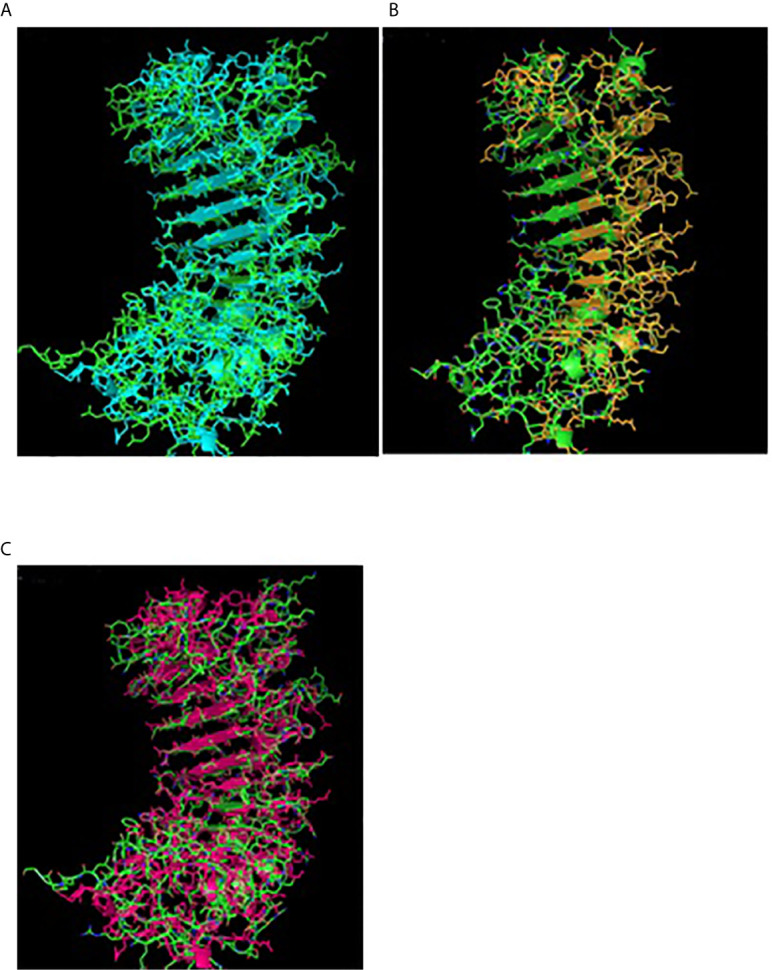
TM alignment of 3D structure of sheep with other ruminants. **(A)** TM alignment of the 3D structure of sheep and goat CD14 molecules. Green, Sheep CD14 molecule, Cyan, Goat CD14 molecule. **(B)** TM alignment of the 3D structure of sheep and cattle CD14 molecules. Green, Sheep CD14 molecule, Orange, Cattle CD14 molecule. **(C)** TM alignment of the 3D structure of sheep and buffalo CD14 molecules; Green, Sheep CD14 molecule; Pink, Buffalo CD14 molecule.

**Figure 5:**
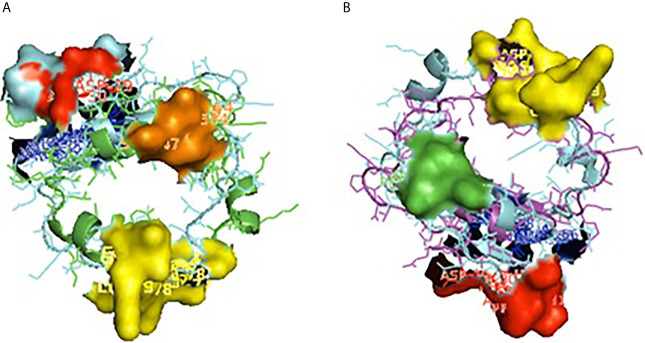
Depiction of receptor binding site for CD14. **(A)** Pocket or groove (LPS binding site) for TM aligned CD14 sheep and CD14 buffalo. **(B)** Pocket or groove (LPS binding site) for TM aligned CD14 sheep and CD14 goat.

A 3-D model of the CD14 molecule of sheep was predicted to be a horseshoe shaped structure, with alternating alpha helix and beta chains in a swiss model (**Figure 3A**), pymol view with LPS binding site (**Figure 3B**), surface view with LPS binding site (**Figure 3C**). **Figure 3D** depicts the groove or pathogen binding site of ovine CD14 surface view. Predicted protein of CD14 molecule revealed a bent solenoid containing a hydrophobic amino terminal pocket. Alignment of 3D structure of ovine CD14 with goat (**Figure 4A**), cattle (**Figure 4B**), and buffalo (**Figure 4C**) have been depicted. The variability in the structure of the pocket of CD14 and alternative rim residues are believed to be important for LPS binding and cell activation, leading to variation in innate immunity across the species. Pockets or grooves (LPS binding site) for TM aligned CD14 sheep and CD14 buffalo have been depicted in **Figure 5A**. Similarly **Figure 5B** depicts pathogen binding sites for ovine CD14 with that of goat CD14. The amino terminal pocket containing grooves are involved in ligand binding (**Figure 9C**). The CD14 receptor exists as a dimer with the help of the leucine zipper in its biologically active form. It had been observed that the amino terminal pocket for ruminant CD14 with grooves is the site for ligand binding, which is the most important factor for CD14. The rim residues of the CD14 hydrophobic pocket are the primary determinants for binding with LPS, or other acylated CD14 ligands. Positively charged residues as K71 and R72 are present on the rim and R80, K87, and R92 are present just outside the rim, whereas hydrophobic residues as W45, F49, V52, F69, Y82, and L89 encircle the rim. These residues were observed to be the primary determinant for ligand binding.

### Comparison of Ovine CD14 With Goat

Small ruminants such as sheep and goats were observed to have significant differences in susceptibility to commonly occurring diseases. Leucine content was observed to be higher in sheep CD14 compared to that of goats. CD14 derived peptide of sheep were observed to have comparatively less GC content, but more leucine content percentage. The number of LRR in buffaloes was observed to be lower in buffalo compared to cattle (**Table 2**). The GPI anchor was present at 353^rd^ position in buffalo, in contrast to 345^th^ position of cattle. The GPI anchor is important for the membranous form of the CD14 gene. 353^rd^ position implies greater availability of CD14.

**Figure 6:**
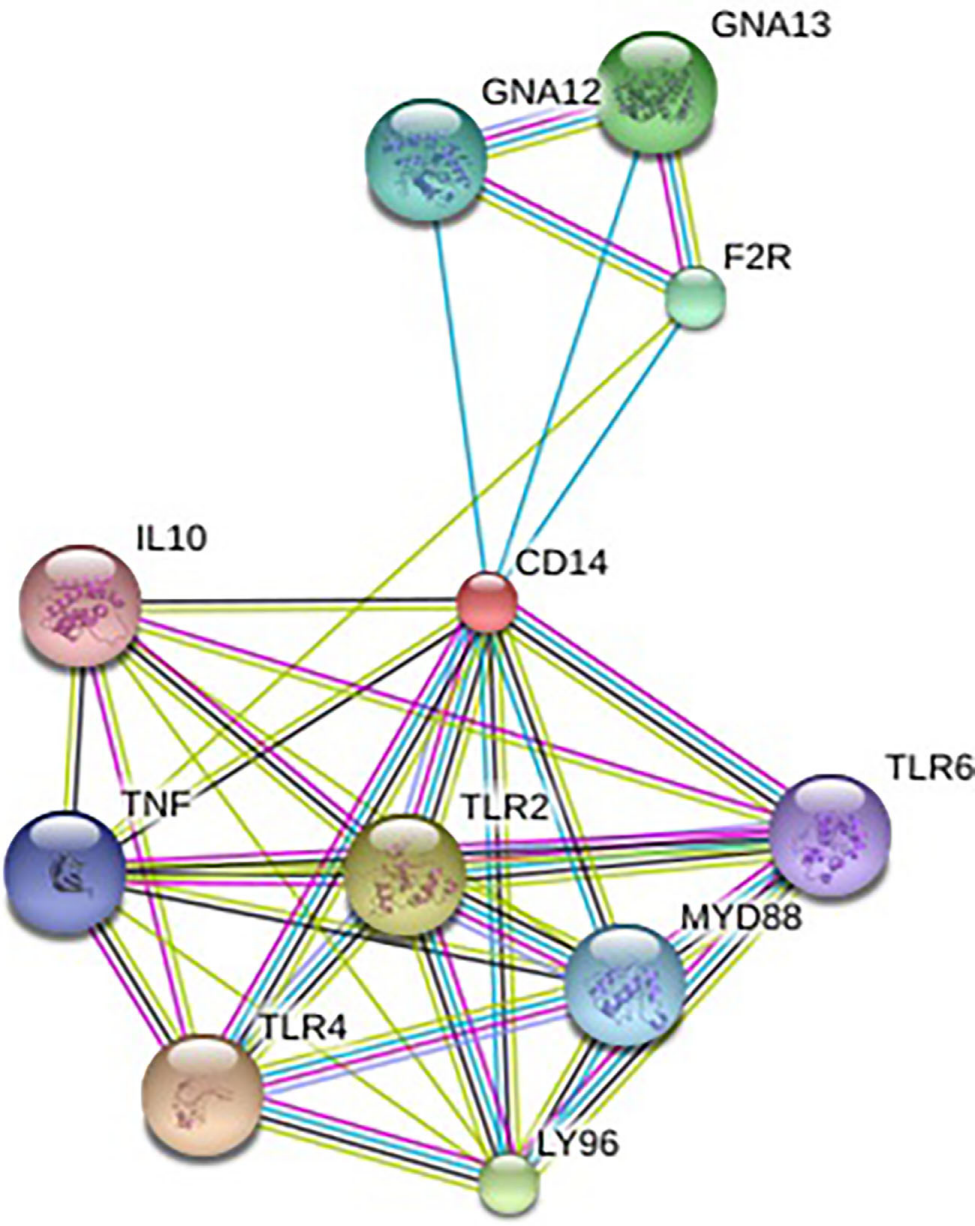
String network-Molecular interaction of ovine CD14 molecule with other related molecules.

**Figure 7:**
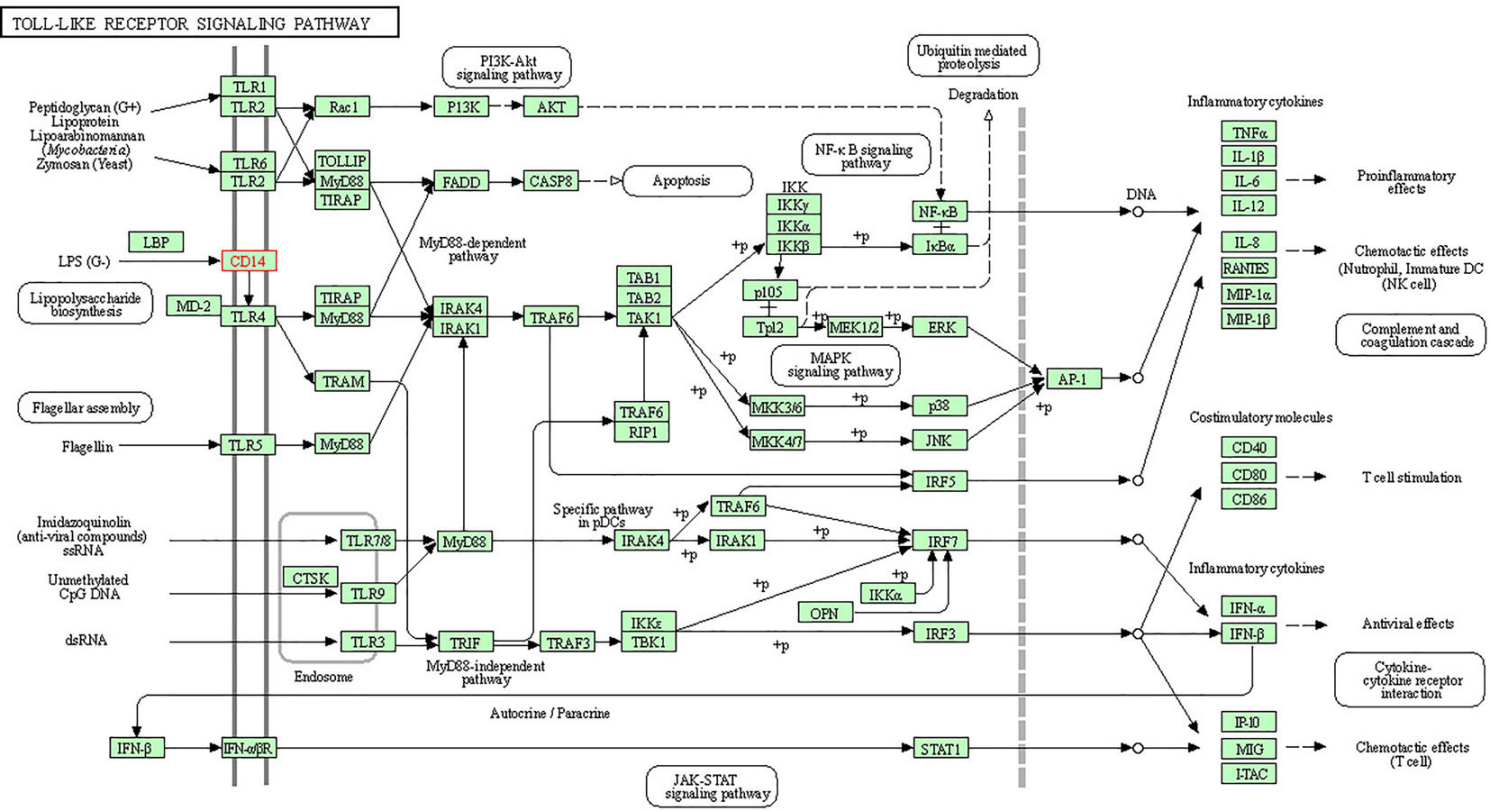
KEGG analysis depicting biochemical pathway involved in CD14 molecules.

Alignment of CD14 (3 D structure) of sheep/goat, sheep/cattle, and sheep/buffalo have been depicted in **Figures 4A–C** respectively. LPS binding sites were situated at the rim of the groove of the receptor. Four LPS binding sites have been depicted as four sites as 29^th^ -32^nd^, 44^th^-47^th^, 55^th^-59^th^ and 75^th^ -80^th^ amino acid positions and depicted in red, green, blue, and yellow respectively (**Figures 5A, B**). A comparison of the CD14 structure of sheep/goats was observed to be highly superimposable with a root mean square deviation of 0.23 A ˚. Observation of the CD14 of goats/buffalo was also observed to be greatly superimposable with RMSD being 1.89. The structural differences arising due to nucleotide differences have been depicted. Comparison of CD14 structure of Sheep/buffalo were observed to be highly super imposable with a root mean square deviation of 0.23. String analysis (**Figure 6**) and KEGG analysis (**Figure 7**) depicted that CD14 works in combination with TLR4 and MD2.

### Molecular Phylogeny

**Figure 8:**
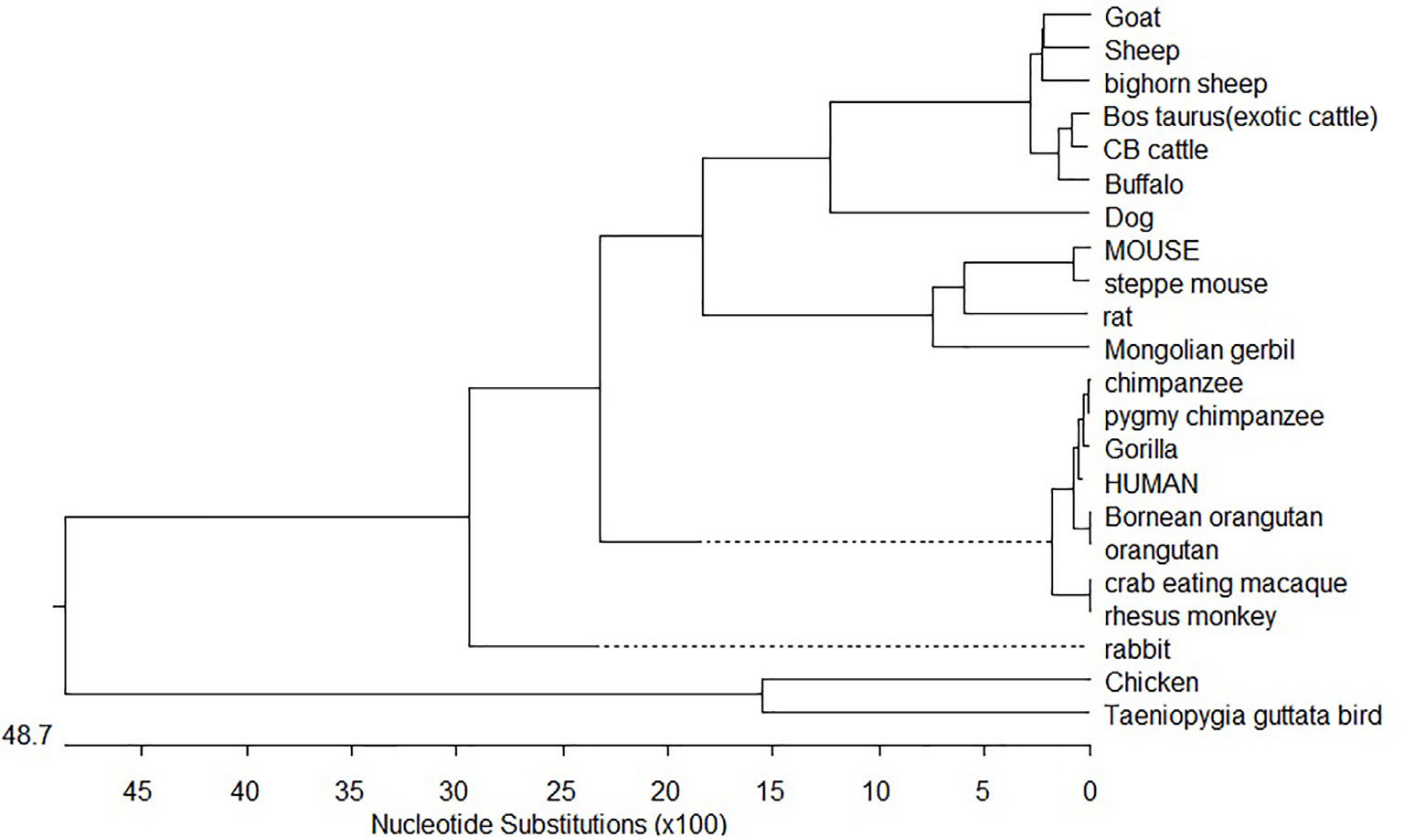
Phylogenetic analysis of mammals and birds with respect to CD14 gene.

Phylogenetic analysis of CD14 DNA sequences strongly suggests that avians diverged first from mammals with 4870 nucleotide substitutions with respect to the CD14 gene (**Figure 8**). Rodents were the next to branch off (**Figure 8**). The phylogenetic tree for ruminants with other mammals and avians of both domestic and wild origin based on the CD14 gene have been depicted in **Figure 8**. Ruminants were clustered together with about 300 nucleotide substitutions. Small ruminants such as sheep and goat were clustered separately from large ruminants such as cattle and buffalo. The closest ancestors for ruminants were found to be dogs. Primates were genetically distant from ruminants at about 2400 nucleotide substitutions. Based on CD14 gene analysis, avians were observed to be genetically distant to mammals at about 4870 nucleotide substitutions. The genetic similarity has been predicted in **Table 1**.

Trans-species polymorphism has been detected in ruminants (**Supplementary Table 2**), where specific alleles were detected for small ruminants (goat and sheep) and large ruminants (cattle and buffalo). Twelve sites were detected (**Supplementary Table 2**) with five synonymous and seven non-synonymous nucleotide substitutions. Non-synonymous substitution exceeding synonymous indicates positive selection. Comparative sequence analysis of cattle and buffalo CD14 has been depicted in **Supplementary Table 2**. Twelve non-synonymous nucleotide substitutions have been depicted in cattle and buffalo CD14 gene. Variations in epidemiological data have been observed in large ruminants with reference to common diseases (**Supplementary Table 3**).

### Assessment of Parasitic Infestation in Sheep

Screening of the faecal egg count (FEC) of the sheep (n=60) lead to study the health condition of the sheep which further prevailed us to divide them into two categories of Non-infected and Infected groups. The mean FECs of the animals are given in the **Table 3**. Direct visualization followed by microscopic examination of the samples (parasites) collected from abomassum reveal the presence of *Haemonchus contortus* for the slaughtered animals.

**Table 3:** Mean FEC of the Non-infected and Infected sheep with *Haemoncus contortus*.

Health Status of the sheep	Mean Faecal Egg Count
Non-infected	50 ± 5.56^a^
Disease	550 ± 9.45^b^

Superscript a and b represent significant differences at P ≤ 0.05.

### Differential mRNA Expression Profile for CD14 Gene With Respect to Non-Infected and Infected Sheep

**Figure 9:**
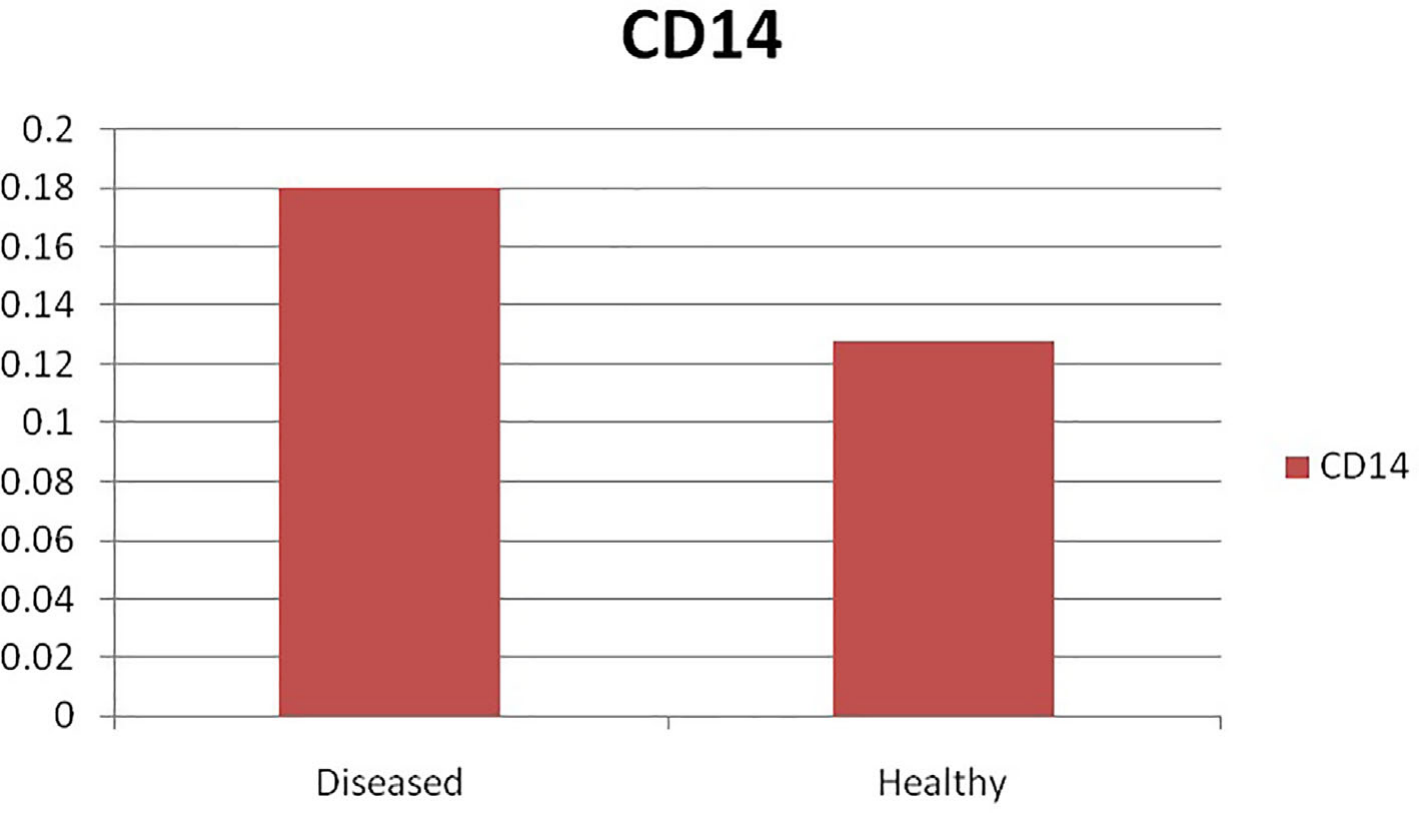
Differential mRNA expression profiling of ovine CD14 with respect to non-infected and infected (Haemonchus infected).

The current study depicts increased expression profile of CD14 in infected (*H. contortus*) infected sheep as depicted in **Figure 9**.

### *In Silico* Study for the Detection of Binding Site of CD14 With *Haemonchus contortus*

**Figure 10:**
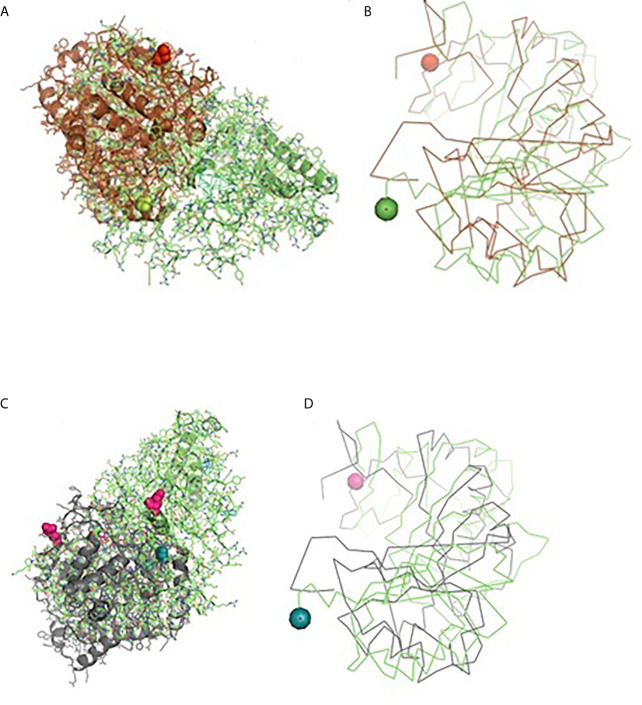
Binding of ovine CD14 with alpha tubulin and beta tubulin of *Haemonchus contortus.* **(A)** Alignment of 3D structure of ovine CD14 molecule (green) with alpha tubulin (brown) of *Haemonchus contortus.* **(B)** Line drawing of binding site of CD14 molecule with alpha tubulin (brown) of *Haemonchus contortus.* **(C)** Alignment of 3D structure of ovine CD14 molecule (green) with beta tubulin (purple) of *Haemonchus contortus.* **(D)** Line drawing of binding site of CD14 molecule (green) with beta tubulin (purple) of *Haemonchus contortus*.

Molecular docking has revealed binding of CD14 with two identified surface molecules of *Haemonchus contortus* as alpha tubulin (**Figures 10A, B**) and beta tubulin(**Figures 10C, D**) with definite binding sites. **Figure 10A** depicts the structural alignment of alpha tubulin of H. contortus with the CD14 gene, and aligned site being depicted as Glu27 (red sphere) and Ala284 (green sphere). **Figure 10B** depicts the line diagram of the alignment of alpha tubulin of H. contortus with ovine CD14. Similarly **Figure 10C** depicts the structural alignment of beta tubulin of H. contortus with the CD14 gene, and the aligned site is depicted as Glu27 (hot pink sphere) and Ala284 (deep teal sphere). **Figure 10D** depicts the line diagram of the alignment of alpha tubulin of H. contortus with ovine CD14. The study reveals distinct binding sites for CD14 with the two most important structural proteins of Haemonchus contortus, alpha tubulin and beta tubulin. This finding indicates the distinct role of CD14 in parasitic immunity. This is the first novel report of the role of CD14 in parasitic immunity, hence comparison was not possible.

### Haematological and Biochemical Parameters With Respect to Non-Infected and Infected Sheep

The haematological parameters were assessed with respect to infected (*H. contortus* infected) vs. non-infected sheep (**Table 4**). Significant differences were observed in Hb gm/dl, PCV%, TEC/cumm, TLC/cumm, Neutrophil %, Eosonophil%, and Lymphocyte%. Better haemoglobin, PCV, and TEC were observed in non-infected sheep in comparison to that of the infected group. However, total leucocyte count was pronounced in infected sheep. A marked significant increase in eosinophil count was observed in infected sheep. A similarly pronounced increase was observed in neutrophil and lymphocytes, indicative of involvement of immune responsiveness.

**Table 4:** Haematological parameters in disease and non-infected sheep.

S.no	Parameter	Disease	Non-infected
1	Hb gm/dl	7.96^b^ ± 0.19	10.37^a^ ± 0.19
2	ESR mm	1.00 ± 0.00	0.90 ± 0.04
3	PCV%	27.84^b^ ± 0.67	36.25^a^ ± 0.64
4	TEC/cumm (10^6^)	3.98^b^ ± 0.05	4.95^a^ ± 0.08
5	TLC/cumm	9200.00^a^ ± 159.23	6587.50^b^ ± 210.81
5	Neutrophil %	53.50^a^ ± 1.00	30.25^b^ ± 0.59
6	Eosonophil%	16. 62^a^ ± 0.49	2.12^b^ ± 0.44
7	Basophil%	0.00	0.00
8	Lymphocyte%	29.12^b^ ± 0.29	60.87^a^ ± 0.29
9	Monocyte%	1.37 ± 0.18	1.87 ± 0.29

The Mean values that have superscript, was showing significant difference between groups. The superscript ‘a’ was showing high mean value than ‘b’. Significant at (p ≤ 0.05).

Significant differences were observed for non-infected and infected sheep in terms of liver function test (**Table 5**) and kidney function test (**Table 6**). Glucose level was observed to be better in non-infected, while Creatinine, uric acid, urea, and BUN level were better in infected sheep. Total protein, albumin, globulin, and albumin: globulin ratio was observed to be better in non-infected sheep. While other reports indicate more in infected sheep (S.G.P.T. S.G.O.T, Alkaline Phosphatase, Total Bilirubin, Direct Bilirubin, Indirect Bilirubin).

**Table 5:** Biochemical parameters (liver function test) in disease and non-infected sheep.

Sr. no	Parameters	Disease	Non-infected
1	Total protein (g/dl)	6.11^b^ ± 0.02	7.77^a^ ± 0.03
2	Albumin (g/dl)	1.92^b^ ± 0.04	2.80^a^ ± 0.03
3	Globulin(g/dl)	4.18^a^ ± 0.06	4.97^b^ ± 0.06
4	Albumin : Globulin	0.46^b^ ± 0.01	0.58^a^ ± 0.01
5	S.G.P.T IU/L	39.75^a^ ± 1.4	32.12^b^ ± 1.0
6	S.G.O.T IU/L	86.87^a^ ± 2.6	68.87^b^ ± 2.7
7	Alkaline Phosphatase IU/L	85.25^a^ ± 3.89	64.87^b^ ± 2.09
8	Total Bilirubin mg/dl	0.67^a^ ± 0.07	0.37^b^ ± 0.03
9	Direct Bilirubin mg/dl	0.30^a^ ± 0.02	0.16^b^ ± 0.01
10	Indirect Bilirubin mg/dl	0.37^a^ ± 0.09014	0.21^b^ ± 0.05

The Mean values that have superscript, was showing significant difference between groups. The superscript ‘a’ was showing high mean value than ‘b’. Significant at (p ≤ 0.05).

**Table 6:** Biochemical parameters (kidney function test) in disease and non-infected sheep.

S.No.	Parameters	Non-infected	Infected
1	Glucose mg/dl	55.50^b^ ± 1.21	68.62^a^ ± 2.33
2	Creatinine mg/dl	1.71^a^ ± 0.05	1.20^b^ ± 0.02
3	Uric acid mg/dl	0.89^a^ ± 0.01	0.76^b^ ± 0.04311
4	Urea mg/dl	48.37^a^ ± 1.2	36.12^b^ ± 1.56
5	BUN mg/dl	23.62^a^ ± 0.73	16.87^b^ ± 0.95
1	Glucose mg/dl	55.50^b^ ± 1.21	68.62^a^ ± 2.33
2	Creatinine mg/dl	1.71^a^ ± 0.05	1.20^b^ ± 0.02

The Mean values that have superscript, was showing significant difference between groups. The superscript ‘a’ was showing high mean value than ‘b’. Significant at (p ≤ 0.05).

## Discussion

Ovine CD14 was characterized and the important domains were identified in comparison to other ruminant species. The GC content of the CD14 gene of humans (62.94%) and dogs (64.07%) was found to be more than ruminants while rodents had less GC content, e.g., rats (56.12%). High GC content was observed to be an inherent characteristic of the CD14 gene. Direct relationship of the length of the coding sequence was observed with the GC content of the gene (75). Species-wise differentiation in GC content may explain the variability in the stability of the CD14 gene among the species. It is known that nucleotide Guanine (G) and cytosine (C) are bounded by triple hydrogen bond in double helix DNA. Thus, it is evident that the GC bond is stronger than the AT bond. Similarly, GC content is reported to have a significant effect on genome functioning and species ecology (75). The importance of GC rich isochores has been predicted. The thermostability, bendability, ability of B-Z transition, and curvature of DNA helix were observed to be correlated positively with GC content (76).

Alignment of the derived peptide sequence of ruminants revealed that leucine residues were mostly conserved across the species (39), except at amino acid position 143. Cysteine residue of CD14 was found to be conserved across ruminant species and other species (**Figure 4**). The CD14 molecule found in ruminants was predicted to contain the higher percentage of leucine (**Table 2**), which is similar to mice (17.66%) and humans (15.5%) (77).

Due to protein folding, certain grooves are developed in CD14, which in turn are responsible for the development of a binding site for ligands. Since the models are developed through homology modelling, 5th disulphide is not visualized in the model. Similar five disulphide bonds were also observed in mice (39) and humans (78). Cys287 is tyrosine moiety in the mouse CD14 sequence, hence from the homology modelling, the fifth disulphide could not be detected.

The crystal structure of CD14 was observed to contain five disulphide bonds both in humans (78) and Mus musculus CD14 molecules (39). Biochemical studies have also detected five disulphide bonds in humans (77). The impact for disulphide bonds differ in human CD14 folding. Out of five disulphide bonds, the first and second disulphide bonds were observed to be indispensable. The third and fourth disulphide bonds were reported to be important, while the fifth or the last (Cys287–Cys333) was dispensable (78).

Disulphide bonds have a greater effect on CD14 structure and integrity, as in the case of mice. The first and second disulphide bonds were observed in the β sheets in the inner concave surface (the “core” structure), whereas the third and fourth disulphide bonds were observed in the loops and helices on the outer surface (the “peripheral” structure).The first disulphide bond is responsible for the structural and functional integrity of CD14 molecule. It was inferred that CD14 folding was not determined by third, fourth, and last disulphide bonds. The substitutional mutation had been observed in the third and fourth disulphide bonds. Cysteine was substituted with alanine, but the function of CD14 is not altered (78).

In ruminants, four LPS binding sites and three LPS signalling sites were predicted. The generous size of the pocket of the CD14 receptor may allow structural variation in the hydrophobic portion of the ligand, however, the hydrophobic bottom and walls of the pocket are rigid. The hydrophilic part of the ligand has structural diversity. This is due to the considerable flexibility of the hydrophilic part of the rim leading to the development of multiple grooves for ligand binding. Similar LPS binding sites and LPS signalling sites were reported in mice (39) and humans (77). The LPS signalling site also overlaps with LRR. It has also been observed that the first LRR (Leucine-rich repeat) overlaps with the LPS-binding site region-I. CD14 is a pattern recognition receptor and is rich in leucine-rich repeat, and CD14 are rich in leucine moiety as evident by high leucine percentage. Ligand specificity or flexibility of the rim of CD14 is important for pathogen binding sites for a wide range of pathogens. There was the report of three, five, four, and five glycosylation sites for N-linked glycosylation in Bos taurus (40), Mus musculus (77, 78), Homo sapiens (77), and Rattus norvegicus (79), respectively. N-linked glycosylation is vital for the molecule as it supports the molecule to be present either in membranous or soluble form. O-linked glycosylation site for ruminant CD14 differs, three in Buffalo and five in both goats and crossbred cattle (**Table 2**), but absent in humans (32). O-linked Glycosylation is needed when hydrophilic clusters of carbohydrates alter the polarity and solubility of protein or protein folding. *Homo sapiens, Mus musculus*, were reported to have 10 LRR each. A trend was observed for the relationship of number of LRR and disease susceptibility. Pathogen recognition is an important phenomenon in any innate immune response. LRR for CD14 in the extracellular domain has a major role in pathogen recognition.

Similar findings as ovine CD14 for GPI anchor were reported in other species like humans and mice. GPI anchor is an important phenomenon for any membranous protein. GPI anchor for CD14 is important for bridging between CD14 and cell surface. In avians, CD14 was observed to be transmembranous, not GPI anchored (80). Amino acid positions responsible for NES are 12, 15, 16, 17, 117, 122, and 127 in ruminant CD14. Since nuclear export signal (NES) aids in export of the CD14 peptide from the nucleus to the cytoplasm *via* the nuclear pore complex (81), there is possibility of impairment of this function in goat CD14. In terms of alpha helix and Beta strands of ovine CD14, there are similarities in mice containing seven of alpha helices, while this differs in the Beta helix. Mice CD14 contains 13 Beta helices in contrast to 11 in ruminants. It seems that Beta helices have a relationship with the innate immunity of theCD14 molecule. Nine Beta strands starting from β2, β4, to β11 overlap with leucine rich repeats (LRRs). LRR in turn have roles inn LPS binding and LPS recognition sites, as already discussed, which forms one of the basis for innate immunity.

Since the CD14 molecule forms its functional form by dimerization, the leucine zipper leads to dimerization. Leucine zippers, a common three-dimensional structural motif (66), were observed to be responsible for gene expression, since these were an integral component of the DNA-binding domain in a variety of transcription factors. The presence of the leucine zipper generates adhesion forces in parallel alpha helices (82) and this region was found to be conserved in ruminant CD14.

Domain linker sequences connect two structural domains and in turn act as a scaffold and prevent unfavourable interactions between folding domains (83).

The CD14 molecule was predicted to be mostly expressed on the cell membrane or cell surface for the ruminants under study, which justifies it to be a receptor molecule. Two forms of CD14, membranous and soluble forms, are in existence (84, 85). Cloned ovine CD14 cDNA in the current study was predicted to be existing in membranous form since it contained GPI anchor (84, 85).

The finding is similar to the study conducted by (39), who studied the crystal structure of the Mus musculus CD14 molecule.

Comparison of ovine CD14 with that of goat has revealed certain differences. Epidemiological studies also depict that sheep were better resistant to goats in terms of infectious and parasitic diseases. GC content was lower in sheep. It has been reported that there is preferential fixation of GC alleles leading to biasness of GC nucleotides over AT during DNA repair. GC content is important with respect to the evolving recombination rate, regulated replication or expression timing, bending ability of DNA, and to B-Z transition, transcriptional efficiency as already explained earlier.

It is evident that a higher mutation frequency was detected in goats (0.56), with mutations detected at 25 amino acid positions, out of which thirteen sites were observed to be thermodynamically unstable. Seven sites were observed to be deleterious in nature.

Mutations detected, such as P13S and P14A in goats, are involved in the domain for signal peptides and were observed to have less structural stability. Mutations were observed such as D30P, D31Q, and D32H, which are responsible for important domains as LPS binding site1, LPS signalling site, and LRR site, where D30P was observed to be deleterious. In site 1 of the LPS binding site, there is a drastic shift of acidic amino acid to basic, such as Aspartic acid to proline, aspartic acid to glutamine, and Aspartic acid to histidine. Since there is a change of polarity, the pathogen binding activity is also adversely effected.

P276I was observed to be thermodynamically unstable, but this site constitutes LRR, which is important for LPS signalling. Thus this mutation may cause depressed function of pathogen binding activity of CD14 of goats in contrast to sheep. Another important site for deleterious mutations is L281C, which is responsible for the domain linker, leucine zipper. K336Q was thermodynamically less stable in goat CD14 and is responsible for the site of domain linker.

A cluster of mutations were observed in goat CD14, which are important for the LRR site, ultimately effecting pathogen binding and signalling, which are G345R, A349D, C350R, S353P,T356V, A364V, L366Y.

MD2 is an important component of TLR4 signalling complex. Members of the MD-2–related lipid-recognition protein super- family contains two beta sheets arranged in ab cup fold generating a centralized hydrophobic LPS-binding cavity (86). CD14 sensitizes cells to LPS through the process of delivering this lipid moeity to MD-2. The hydrophobic pocket is occupied mostly by four acyl chains of the ligand buried inside for the complex of both mouse and human CD14 with MD-2 bound to lipid IVa (87, 88). TLR4/MD-2/LPS homodimeric signalling complex depicts the hydrophobic binding pocket sequestering five of the six fatty acid chains. The rest of the unbound acyl chain lies along the surface of MD-2 and, along with the F126 loop of MD-2, generates a new hydrophobic patch promoting homodimer formation through the association of TLR4/MD-2/LPS complex (19).

LPS-binding pocket (hydrophobic) was predicted at the amino terminal end through a homology based study with 3D structure of CD14 of Mus musculus (39) and Homo sapiens (77). CD14 molecule, α-helices were located at the convex surface, and β strands were located at the concave surface of horse-shoe shaped CD14 structure, represented in **Figures 3**, **5**. N terminal hydrophobic pocket revealed a conservation of pocket size and hydrophobicity. Asparagine ladder with conserved asparagines residue had been observed, which is a structural feature for leucine rich repeats. The variable residues in LRR are hydrophilic and exposed to the concave surface of the horseshoe structure. Some of the similar studies had been conducted earlier in ruminants (53, 55).

CD14 has been considered as an important gene to explore a wide range of open questions in evolutionary ecology, since a wide range of nucleotide variability has been observed with the mutational hot spot detected. It was attempted to identify the Trans species polymorphism (TSP).

TSP generally occurs by passage of alleles from ancestral to descendant species. Neutral alleles may be shared by species which are genetically similar. Balancing selection has been regarded as the major evolutionary force responsible for long-lasting TSP. Similar TSP as identified in ruminants with respect to the CD14 gene have also been identified in the MHC Class II gene (89). Speciation is a complex process which involves TSP, divergence of allelic lineages at the coding sites for both synonymous and non-synonymous mutations. These processes took places millions of years ago (90).

Pronounced expression of CD14 was observed in infected sheep compared to that of non-infected sheep. As per our literature search, it is the first report in sheep. A similarly improved expression profile of CD14 was also detected in mice infected with another parasite as *Schistosoma mansoni*  (91).

Some more studies suggested that CD14^+^ and CD16^+^ cells help to control the parasitic burden and play important roles in resistance to *P. vivax* infection in humans (92). However, in our present study increased expression of CD14 gene in infected sheep may be due to its action as a pattern recognition receptor in parasitic infection. It was reported that parasite-derived E/S product was detected by CD14^bright/^CD16^+^ intermediate monocytes in case of schistosome-infected patients compared to that of uninfected individuals (93). In addition, CD14 has a significant role in regulating the immunity in *S. mansoni*-infected mice, through the induction of both TH1 and TH2 through macrophage M1/M2 phenotype. CD14^bright^CD16^+^ intermediate monocytes are major producer of IL-10 (94). The Th1 and Th2 cell released different kinds of cytokines such as interleukin (IL-2), interferon-gamma (IFN-γ) and tumour necrosis factor-alpha (TNF-α) from TH1 cells and IL-10 produce from Th2 type cells and these cytokines are responsible to stimulates B cell differentiation, production of different immunoglobulin such as IgE, IgG1, IgG4, and IgA, mastocytosis, and eosinophil activation and function (95).

IL-10 leading to eosinophil mobilization, intestinal mast cell accumulation and production of IgE, mucosal mast cell infiltration, intestinal eosinophilia, elevated serum IgE, increased level of parasite specific IgG-1 which are responsible to eradicate worms (96).

Similar studies that correspond to our finding shows a marked increase in blood eosinophil and abomasal tissue, degranulation of eosinophil and it releases some peroxidases and basic protein and cationic protein which are cytotoxic to helminth (9). Similar studies of caprine CD14 had been conducted by us earlier with focus on evolution and structural analysis (97).

## Conclusion

CD 14, an important gene for innate immunity, has revealed immense variability between species, which may lead to variability in resistance to diseases. This variability in disease resistance may be due to variability in ligand binding ability of the CD14 molecule. The greater susceptibility of goat compared to sheep, and greater susceptibility of cattle compared to buffalo revealed mutations in LPS binding site of the goat/cattle CD14 molecule, which causes altered polarity, decreased thermodynamic stability, and the deleterious mutation. Since hypervariable sites or mutational hot spots have been detected in the CD14 gene, due to greater genetic variability, evolutionary study is very promising. Phylogenetic analysis reveals that ruminants were evolutionary closer, but transpecies polymorphism has been detected among the ruminants. The distinct role of CD14 in antiparasitic immunity is reported here for the first time through *in silico* molecular docking and later confirmed through differential mRNA expression profile with QPCR for non-infected and infected samples.

## Data Availability Statement

The datasets presented in this study can be found in online repositories. The names of the repository/repositories and accession number(s) can be found in the article/**Supplementary Material**.

## Ethics Statement

The animal study was reviewed and approved by Institutional Animal Ethics committee, West Bengal University of Animal and Fishery Sciences.

## Author Contributions

KR has conducted the experimental work, collected samples, performed acquisition of data, drafted the article.*ArP has conceptualized and designed the work, conducted research, analysed and interpreted the data, and drafted the article. SaB has conducted the experimental work. AbP has conducted the bioinformatics analysis and its interpretations.SCM has guided through the experiments on parasitology. SuB has conceptualized the work, guided through the biochemical analysis. All authors contributed to the article and approved the submitted version.

## Conflict of Interest

The authors declare that the research was conducted in the absence of any commercial or financial relationships that could be construed as a potential conflict of interest.

## Supplementary Material

Supplementary Table 1 List of mammals & avian under present study analysed for CD14 sequences.

Supplementary Table 2 Trans species polymorphism detected from molecular evolutionary study of CD14 gene for the ruminants.

Supplementary Table 3 Epidemiological data (in percentage) for large ruminants with reference to common diseases.
