# Supplementary material for: Ovine CD14- an Immune Response Gene Has a Role Against Gastrointestinal Nematode Haemonchus contortus—A Novel Report
Source: Front Immunol. 2021 Jul 16;12:664877. doi: 10.3389/fimmu.2021.664877 (PMC8324245; doi:10.3389/fimmu.2021.664877)
Supplement: Supplementary Table 1 — List of mammals & avian under present study analysed for CD14 sequences. [file Table_1.doc]

Supplementary Table 1: List of mammals & avian under present study

| Orders | IUCN Red List of Threatened Species | Species | | **Gene bank Accession no.**  **CD14** |
| --- | --- | --- | --- | --- |
| **Mammals** | | | | |
| Artiodactyla |  | Sheep | *Ovies aries* | KY110723* |
|  |  | Bighorn sheep | *Ovis canadensis* | M37310 |
|  |  | Buffalo | *Bubalus bubalis* | DQ457089** |
|  |  | Cattle (CB) | *Bos taurus X Bos indicus* | GU368102** |
|  |  | Goat | *Capra hircus* | DQ457090** |
| Camivora |  | Dog | *Canis familiaris* | EU263365 |
| Lagomorpha | Near threatened | Rabbit | *Oryctolagus cuniculus* | M85233.1 |
| Primates | Endangered | Chimpanzee | *Pan troglodytes* | NM_001130466.1 |
|  |  | Rhesus monkey | *Macaca mulatta* | NM_001130433 |
|  | Near threatened | Guinea baboon | *Papio papio* | DQ482129 |
|  | Critically endangered | Western Gorilla | *Gorilla gorilla* | AB446508 |
|  |  | Crab-eating macaque | *Macaca fascicularis* | AB446510 |
|  | Endangered | Pygmy chimpanzee | *Pan paniscus* | AB446507 |
|  | Endangered | Bornean orangutan | *Pongo pygmaeus* | AB446509 |
|  |  | Olive baboon | *Papio anubis* | DQ340390 |
|  |  | Human | *Homo sapiens* | NM_000591 |
| Rodentia |  | Mice | *Mus musculus* | NM_009841 |
|  |  | Rat | *Rattus norvegicus* | NM_021744.1 |
|  |  | Mongolian gerbil | *Meriones unguiculatus* | AB429405 |
|  |  | Steppe mouse | *Mus spicilegus* | AB039063 |
| **Avian** | | | | |
| Galliformes |  | Chicken | *Gallus gallus* | NM_204359 |
| Passeriformes |  | Zebra finch | *Taeniopygia guttata* | XM_002196131 |

* Sequenced in the present study

** Sequenced in our earlier study

**Supplementary Table 2: Trans species polymorphism detected from molecular evolutionary study of CD14 gene for the ruminants**

| **Nucleotide positions** | **Nucleotide in small ruminants**  **(Sheep & Goat)** | **Nucleotide in large ruminants**  **(Cattle and Buffalo)** | **Resulting amino acid change in small ruminants** | **Resulting amino acid change in large ruminants** |
| --- | --- | --- | --- | --- |
| 102 | **C** | **T** | **Synm** | |
| 181 | **C** | **A** | **R** | **S** |
| 194 | **A** | **G** | **H** | **R** |
| 204 | **C** | **A** | **D** | **E** |
| 223 | **A** | **G** | **N** | **D** |
| 229 | **G** | **A** | **D** | **N (Buffalo)**  **T (Cattle)** |
| 384 | **T** | **C** | **Synm** | |
| 518 | **C** | **T** | **A** | **V** |
| 639 | **G** | **A** | **Synm** | |
| 873 | **A** | **G** | **Synm** | |
| 882 | **G** | **C** | **Synm** | |
| 962 | **T** | **A** | **V** | **D** |

Supplementary Table 3: Epidemiological data (in percentage) for large ruminants with reference to common diseases.

| **Sl no.** | **Classification**  **of disease** | **Cattle** | | | **Buffalo** | | |
| --- | --- | --- | --- | --- | --- | --- | --- |
|  |  | **Summer** | **Winter** | Round the  Year | **Summer** | **Winter** | **Round**  **the year** |
| **1** | **Systemic disease**  **Includes**  **Infectious disease** | 62.14 | 30.84 | 92.99 | 7.00 | - | 7.00 |
| **2** | **Parasitic disease** | 51.54 | 41.23 | 92.78 | 4.12 | 3.09 | 7.21 |
|  | Stephanofilariasis | 56.60 | 37.73 | 94.34 | 3.74 | 1.88 | 5.66 |
|  | Ecto-parasitic inf | 42.85 | 42.85 | 85.71 | 14.28 | - | 14.28 |
| **3.** | **Localized eye/**  **ear infection** | 42.10 | 31.57 | 73.68 | 10.52 | 15.78 | 26.31 |
